# 2,6-Dibromo-4-chloro­aniline

**DOI:** 10.1107/S1600536812023331

**Published:** 2012-05-26

**Authors:** Umar Sharif Ali, Waseeq Ahmad Siddiqui, Adnan Ashraf, M. Nawaz Tahir

**Affiliations:** a University of Sargodha, Department of Chemistry, Sargodha, Pakistan; b University of Sargodha, Department of Physics, Sargodha, Pakistan

## Abstract

The title compound, C_6_H_4_Br_2_ClN, is almost planar (r.m.s. deviation = 0.024 Å) and two intra­molecular N—H⋯Br hydrogen bonds generate *S*(5) rings. In the crystal, N—H⋯Br hydrogen bonds link the mol­ecules into chains propagating in [010].

## Related literature

For related structures, see: Schlemper & Konnert (1967 ▶): Takazawa *et al.* (1989 ▶). For the synthesis, see: Harrison *et al.* (1981 ▶). For graph-set notation, see: Bernstein *et al.* (1995 ▶).
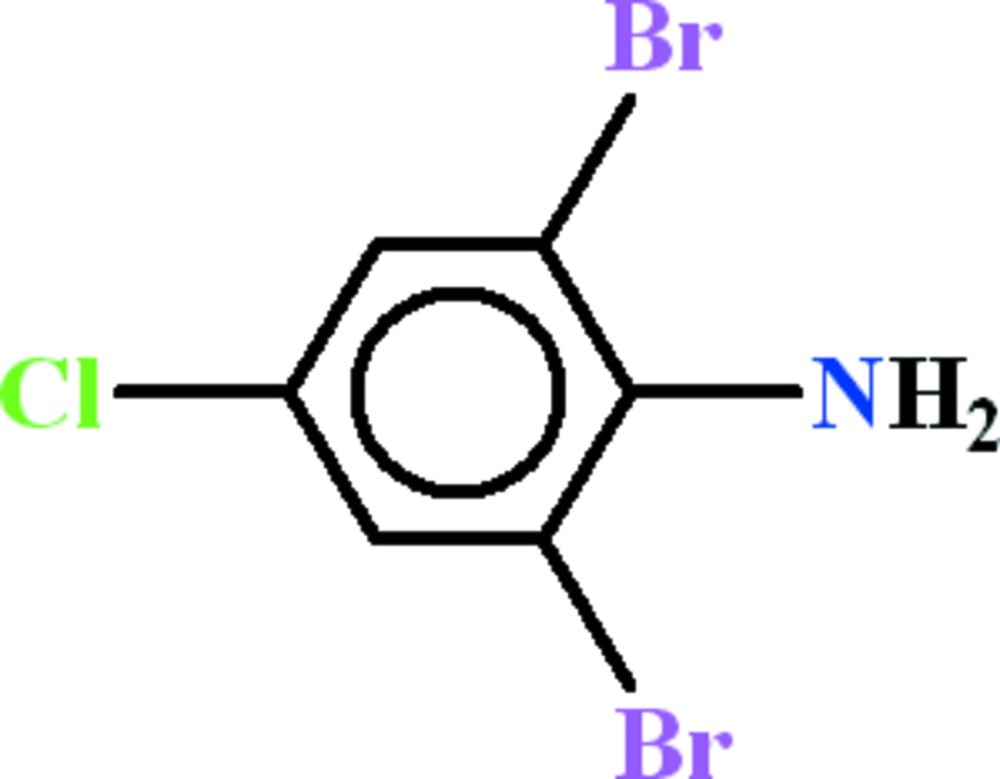


## Experimental

### 

#### Crystal data

C_6_H_4_Br_2_ClN
*M* *_r_* = 285.37
Monoclinic, 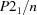

*a* = 13.3132 (7) Å
*b* = 3.9387 (2) Å
*c* = 16.5476 (9) Å
β = 112.318 (2)°
*V* = 802.70 (7) Å^3^
*Z* = 4
Mo *K*α radiation
μ = 10.35 mm^−1^
*T* = 296 K
0.35 × 0.15 × 0.12 mm

#### Data collection

Bruker Kappa APEXII CCD diffractometer
Absorption correction: multi-scan (*SADABS*; Bruker, 2005 ▶) *T*_min_ = 0.170, *T*_max_ = 0.292
6640 measured reflections
1900 independent reflections
1429 reflections with *I* > 2σ(*I*)
*R*_int_ = 0.028

#### Refinement

*R*[*F* ^2^ > 2σ(*F* ^2^)] = 0.024
*wR*(*F* ^2^) = 0.056
*S* = 1.02
1900 reflections
92 parameters
H-atom parameters constrained
Δρ_max_ = 0.43 e Å^−3^
Δρ_min_ = −0.36 e Å^−3^

### 

Data collection: *APEX2* (Bruker, 2009 ▶); cell refinement: *SAINT* (Bruker, 2009 ▶); data reduction: *SAINT*; program(s) used to solve structure: *SHELXS97* (Sheldrick, 2008 ▶); program(s) used to refine structure: *SHELXL97* (Sheldrick, 2008 ▶); molecular graphics: *ORTEP-3 for Windows* (Farrugia, 1997 ▶) and *PLATON* (Spek, 2009 ▶); software used to prepare material for publication: *WinGX* (Farrugia, 1999 ▶) and *PLATON*.

## Supplementary Material

Crystal structure: contains datablock(s) global, I. DOI: 10.1107/S1600536812023331/hb6810sup1.cif

Structure factors: contains datablock(s) I. DOI: 10.1107/S1600536812023331/hb6810Isup2.hkl

Supplementary material file. DOI: 10.1107/S1600536812023331/hb6810Isup3.cml

Additional supplementary materials:  crystallographic information; 3D view; checkCIF report

## Figures and Tables

**Table 1:** Hydrogen-bond geometry (Å, °)

*D*—H⋯*A*	*D*—H	H⋯*A*	*D*⋯*A*	*D*—H⋯*A*
N1—H1*A*⋯Br2	0.86	2.64	3.067 (2)	112
N1—H1*A*⋯Br2^i^	0.86	2.91	3.380 (3)	117
N1—H1*B*⋯Br1	0.86	2.67	3.099 (3)	112

Symmetry code: (i) 
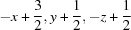
.

**Fig. 1.**
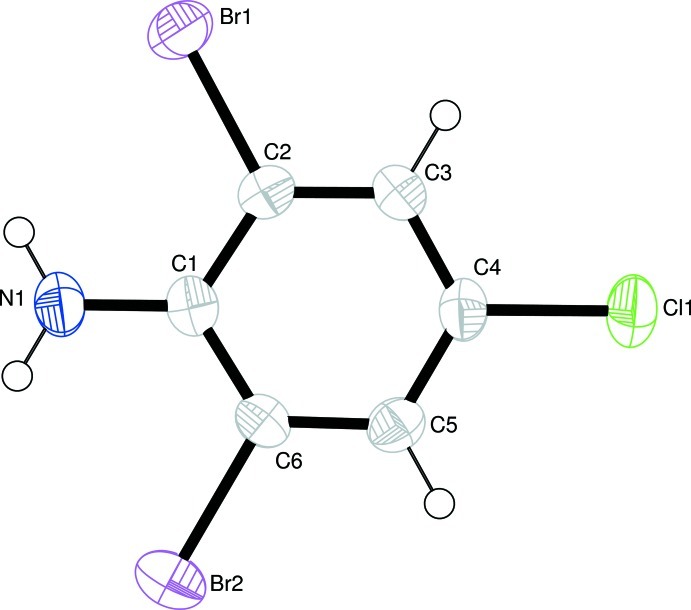
View of the title compound with displacement ellipsoids drawn at the 50% probability level.

**Fig. 2.**
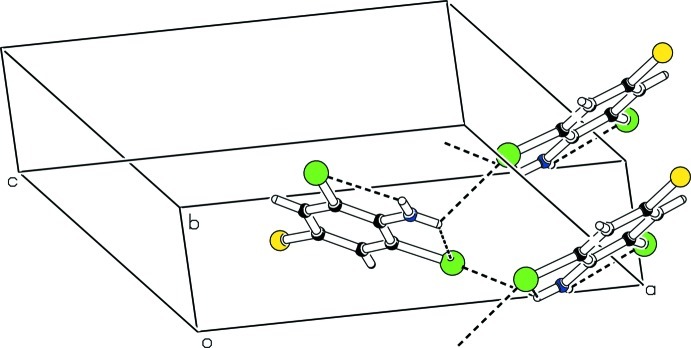
The partial packing (PLATON; Spek, 2009), which shows that molecules form C(2) chains extending along [0 1 0] direction.

## supplementary crystallographic information

### Comment

The title compound (I), (Fig. 1) has been synthesized as a pre-cursor. The crystal structures of 4-chloroaniline (Takazawa *et al.*, 1989), 2,4, 6-tribromoaniline (Schlemper & Konnert, 1967) have been published which are related to (I).

The molecule as a whole is almost planar with r. m. s. deviation of 0.0242 Å. In (I), there exist intramolecular H-bonding of N—H···Br type to form two S(5) rings (Bernstein *et al.*, 1995). The molecules are connected along the *b*-axis due to H-bondings of N—H···Br type (Table 1, Fig. 2). There does not exist any kind of π-interaction.

### Experimental

The title compound has been synthesized from the 4-chloroaniline using the method of Harrison, *et al.*, 1981.

m. p. 352–354 K.

### Refinement

The H-atoms were positioned geometrically at C—H = 0.93 and N—H = 0.86 Å, respectively and included in the refinement as riding with *U*_iso_(H) = *xU*_eq_(C, N), where *x* = 1.2 for all H atoms.

### Crystal data

**Table:**

C_6_H_4_Br_2_ClN	*F*(000) = 536
*M**_r_* = 285.37	*D*_x_ = 2.361 Mg m^−^^3^
Monoclinic, *P*2_1_/*n*	Mo *K*α radiation, λ = 0.71073 Å
Hall symbol: -P 2yn	Cell parameters from 1429 reflections
*a* = 13.3132 (7) Å	θ = 2.5–27.9°
*b* = 3.9387 (2) Å	µ = 10.35 mm^−^^1^
*c* = 16.5476 (9) Å	*T* = 296 K
β = 112.318 (2)°	Needle, dark brown
*V* = 802.70 (7) Å^3^	0.35 × 0.15 × 0.12 mm
*Z* = 4	

### Data collection

**Table:**

Bruker Kappa APEXII CCD diffractometer	1900 independent reflections
Radiation source: fine-focus sealed tube	1429 reflections with *I* > 2σ(*I*)
Graphite monochromator	*R*_int_ = 0.028
Detector resolution: 7.60 pixels mm^-1^	θ_max_ = 27.9°, θ_min_ = 2.5°
ω scans	*h* = −17→17
Absorption correction: multi-scan (*SADABS*; Bruker, 2005)	*k* = −3→5
*T*_min_ = 0.170, *T*_max_ = 0.292	*l* = −21→21
6640 measured reflections	

### Refinement

**Table:**

Refinement on *F*^2^	Secondary atom site location: difference Fourier map
Least-squares matrix: full	Hydrogen site location: inferred from neighbouring sites
*R*[*F*^2^ > 2σ(*F*^2^)] = 0.024	H-atom parameters constrained
*wR*(*F*^2^) = 0.056	*w* = 1/[σ^2^(*F*_o_^2^) + (0.0225*P*)^2^ + 0.3103*P*] where *P* = (*F*_o_^2^ + 2*F*_c_^2^)/3
*S* = 1.02	(Δ/σ)_max_ = 0.001
1900 reflections	Δρ_max_ = 0.43 e Å^−^^3^
92 parameters	Δρ_min_ = −0.36 e Å^−^^3^
0 restraints	Extinction correction: *SHELXL97* (Sheldrick, 2008), Fc^*^=kFc[1+0.001xFc^2^λ^3^/sin(2θ)]^-1/4^
Primary atom site location: structure-invariant direct methods	Extinction coefficient: 0.0048 (5)

### Special details

**Table:**

Geometry. Bond distances, angles *etc*. have been calculated using the rounded fractional coordinates. All su's are estimated from the variances of the (full) variance-covariance matrix. The cell e.s.d.'s are taken into account in the estimation of distances, angles and torsion angles
Refinement. Refinement of *F*^2^ against ALL reflections. The weighted *R*-factor *wR* and goodness of fit *S* are based on *F*^2^, conventional *R*-factors *R* are based on *F*, with *F* set to zero for negative *F*^2^. The threshold expression of *F*^2^ > σ(*F*^2^) is used only for calculating *R*-factors(gt) *etc*. and is not relevant to the choice of reflections for refinement. *R*-factors based on *F*^2^ are statistically about twice as large as those based on *F*, and *R*- factors based on ALL data will be even larger.

### Fractional atomic coordinates and isotropic or equivalent isotropic displacement parameters (Å^2^)

**Table:**

	*x*	*y*	*z*	*U*_iso_*/*U*_eq_	
Br1	0.43969 (2)	0.63712 (8)	0.36884 (2)	0.0452 (1)	
Br2	0.63382 (2)	0.13268 (8)	0.14100 (2)	0.0465 (1)	
Cl1	0.21601 (6)	0.6350 (2)	0.01323 (5)	0.0528 (3)	
N1	0.61671 (18)	0.3035 (7)	0.31614 (15)	0.0447 (8)	
C1	0.5233 (2)	0.3766 (6)	0.24594 (17)	0.0310 (8)	
C2	0.4327 (2)	0.5295 (7)	0.25508 (16)	0.0312 (8)	
C3	0.3397 (2)	0.6082 (7)	0.18522 (17)	0.0333 (8)	
C4	0.3335 (2)	0.5335 (7)	0.10201 (18)	0.0351 (8)	
C5	0.4205 (2)	0.3896 (7)	0.08856 (17)	0.0355 (8)	
C6	0.5129 (2)	0.3157 (6)	0.15999 (17)	0.0301 (8)	
H1A	0.67145	0.21719	0.30798	0.0537*	
H1B	0.62055	0.34404	0.36830	0.0537*	
H3	0.28153	0.71053	0.19381	0.0399*	
H5	0.41666	0.34358	0.03233	0.0426*	

### Atomic displacement parameters (Å^2^)

**Table:**

	*U*^11^	*U*^22^	*U*^33^	*U*^12^	*U*^13^	*U*^23^
Br1	0.0471 (2)	0.0548 (2)	0.0351 (2)	−0.0064 (1)	0.0173 (1)	−0.0081 (1)
Br2	0.0434 (2)	0.0423 (2)	0.0610 (2)	0.0059 (1)	0.0281 (2)	−0.0024 (2)
Cl1	0.0439 (4)	0.0637 (6)	0.0373 (4)	0.0116 (4)	0.0002 (3)	0.0030 (4)
N1	0.0297 (12)	0.0628 (18)	0.0369 (13)	0.0035 (12)	0.0072 (10)	0.0046 (12)
C1	0.0284 (13)	0.0255 (14)	0.0371 (13)	−0.0050 (12)	0.0103 (11)	0.0035 (12)
C2	0.0352 (14)	0.0284 (15)	0.0312 (13)	−0.0071 (12)	0.0141 (12)	−0.0026 (11)
C3	0.0294 (13)	0.0317 (16)	0.0384 (14)	0.0017 (12)	0.0125 (12)	0.0000 (12)
C4	0.0332 (14)	0.0315 (16)	0.0333 (14)	0.0024 (12)	0.0043 (12)	0.0020 (12)
C5	0.0412 (15)	0.0334 (16)	0.0320 (13)	−0.0017 (13)	0.0141 (12)	−0.0033 (12)
C6	0.0299 (13)	0.0229 (15)	0.0391 (14)	0.0004 (11)	0.0150 (11)	0.0009 (12)

### Geometric parameters (Å, º)

**Table:**

Br1—C2	1.897 (3)	C1—C2	1.407 (4)
Br2—C6	1.896 (3)	C2—C3	1.371 (4)
Cl1—C4	1.738 (3)	C3—C4	1.380 (4)
N1—C1	1.372 (4)	C4—C5	1.381 (4)
N1—H1B	0.8600	C5—C6	1.375 (4)
N1—H1A	0.8600	C3—H3	0.9300
C1—C6	1.396 (4)	C5—H5	0.9300
			
C1—N1—H1B	120.00	Cl1—C4—C3	119.2 (2)
H1A—N1—H1B	120.00	Cl1—C4—C5	119.9 (2)
C1—N1—H1A	120.00	C4—C5—C6	118.7 (2)
C2—C1—C6	115.2 (2)	Br2—C6—C1	118.2 (2)
N1—C1—C6	122.3 (3)	Br2—C6—C5	118.5 (2)
N1—C1—C2	122.5 (2)	C1—C6—C5	123.3 (3)
Br1—C2—C1	118.78 (19)	C2—C3—H3	120.00
Br1—C2—C3	118.3 (2)	C4—C3—H3	120.00
C1—C2—C3	122.9 (2)	C4—C5—H5	121.00
C2—C3—C4	119.1 (3)	C6—C5—H5	121.00
C3—C4—C5	120.8 (3)		
			
N1—C1—C2—Br1	−0.3 (4)	Br1—C2—C3—C4	179.6 (2)
N1—C1—C2—C3	179.0 (3)	C1—C2—C3—C4	0.4 (4)
C6—C1—C2—Br1	−178.01 (19)	C2—C3—C4—Cl1	−179.7 (2)
C6—C1—C2—C3	1.2 (4)	C2—C3—C4—C5	−1.7 (4)
N1—C1—C6—Br2	−1.1 (3)	Cl1—C4—C5—C6	179.2 (2)
N1—C1—C6—C5	−179.4 (3)	C3—C4—C5—C6	1.3 (4)
C2—C1—C6—Br2	176.68 (19)	C4—C5—C6—Br2	−177.9 (2)
C2—C1—C6—C5	−1.6 (4)	C4—C5—C6—C1	0.5 (4)

### Hydrogen-bond geometry (Å, º)

**Table:**

*D*—H···*A*	*D*—H	H···*A*	*D*···*A*	*D*—H···*A*
N1—H1*A*···Br2	0.86	2.64	3.067 (2)	112
N1—H1*A*···Br2^i^	0.86	2.91	3.380 (3)	117
N1—H1*B*···Br1	0.86	2.67	3.099 (3)	112

Symmetry code: (i) −*x*+3/2, *y*+1/2, −*z*+1/2.
